# Advancements in Therapeutic Approaches for Degenerative Tendinopathy: Evaluating Efficacy and Challenges

**DOI:** 10.3390/ijms252111846

**Published:** 2024-11-04

**Authors:** Vivek Kumar Morya, Hamzah Shahid, Jun Lang, Mi Kyung Kwak, Sin-Hye Park, Kyu-Cheol Noh

**Affiliations:** 1 Hallym University Dongtan Sacred Heart Hospital, Hwaseong-si 18450, Republic of Korea; moryavivek@hallym.ac.kr (V.K.M.); langjun1994@gmail.com (J.L.);; 2 School of Medicine, Hallym University, Chuncheon 24252, Republic of Korea; 3 Department of Food Science & Nutrition, Hallym University, Chuncheon 24252, Republic of Korea; 4 Hallym University Sacred Heart Hospital, Anyang-si 14068, Republic of Korea

**Keywords:** tendinopathy, regenerative medicine, therapeutic advancements

## Abstract

Degenerative tendinopathy results from the accumulation of minor injuries following unsuccessful tendon repair during acute tendon injuries. The process of tendon repair is prolonged and varies between individuals, making it susceptible to reinjury. Moreover, treating chronic tendinopathy often requires expensive and extensive rehabilitation, along with a variety of combined therapies to facilitate recovery. This condition significantly affects the quality of life of affected individuals, underscoring the urgent need for more efficient and cost-effective treatment options. Although traditional treatments have improved significantly and are being used as substitutes for surgical interventions, the findings have been inconsistent and conflicting. This review aims to clarify these issues by exploring the strengths and limitations of current treatments as well as recent innovations in managing various forms of degenerative tendinopathy.

## 1. Introduction

**Figure 1:**
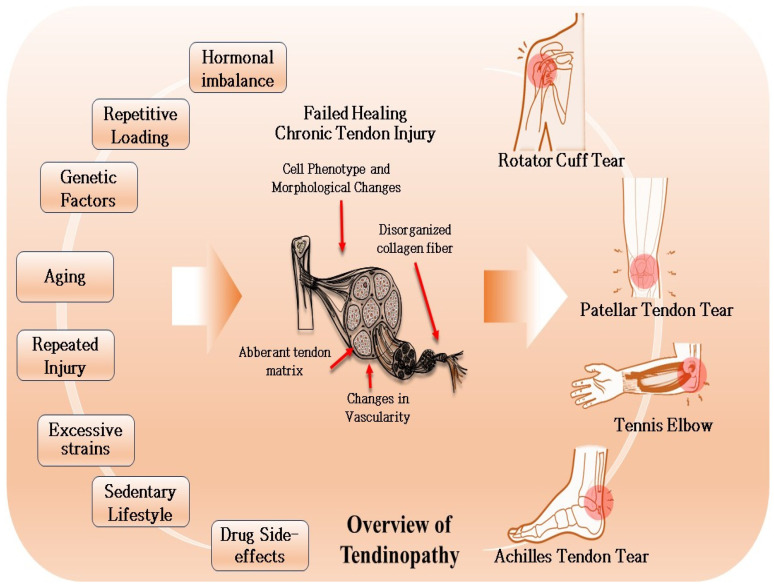
Tendinopathy associated changes: The figure provides an overview of the multifactorial causes and pathological changes involved in tendinopathy. Intrinsic factors such as genetic predisposition, aging, and hormonal imbalances, along with extrinsic factors like repetitive mechanical loading, sedentary lifestyle, and drug side effects contribute to the onset and progression of chronic tendon injuries. The degenerative process is marked by a continuum of changes, including failed healing, alterations in cell phenotype, and structural disruptions such as disorganized collagen fibers, altered tendon matrix, and increased or abnormal vascularity. These pathological changes are implicated in various conditions like rotator cuff tear, patellar tendon tear, tennis elbow, and Achilles tendon tear.

Degenerative tendinopathy (DT) encompasses a broad spectrum of chronic tendon disorders characterized by persistent local pain and stiffness in the affected area, potentially progressing to tendon rupture (Figure 1). Although exact incidence rates are challenging to determine, it is estimated that tendinopathy accounts for 30% of musculoskeletal-associated consultations [1]. Tendon overloading can exacerbate these conditions, leading to progressive degeneration [2].

Tendons are essential in the musculoskeletal system as they transmit forces between muscles and bones. However, overloading can impair tendon function, resulting in chronic pain, a key feature of tendinopathy [1,2,3]. Excessive loading is a major cause of tendon injury due to the tendon’s slow metabolism, which enables it to endure high stress but also slows healing [4]. While physical rehabilitation can help reduce symptoms, some patients do not benefit from conventional treatments because of various intrinsic factors (e.g., age, genetics) and extrinsic factors (e.g., activity level, treatment adherence). These patients often struggle to return to normal life [5]. Advanced imaging techniques, such as ultrasound and MRI, are vital for diagnosing tendon-related disorders. To better understand tendon pathology, researchers have created animal models that mimic the histological features of both healthy and tendinopathic tendons. These models are crucial for investigating the molecular and cellular mechanisms underlying degenerative tendon conditions and for developing more effective therapies [6].

Common cases of degenerative tendinopathy (DT) include Achilles tendinopathy (AT), rotator cuff tendinopathy (RCT), patellar tendinopathy (PT), and lateral elbow tendinopathy (LET) [7]. Risk factors for tendinopathy are categorized as intrinsic (age, gender, genetics, weight, health conditions) or extrinsic (repetitive loading, medications, socioeconomic factors) [8]. Among these, aging and muscular overuse are significant contributors to DT [9].

**Figure 2:**
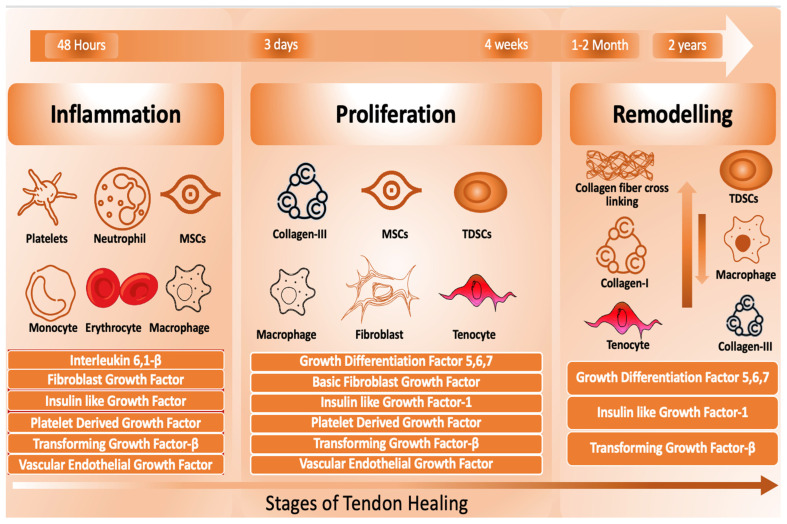
Stages of tendon healing: This figure delineates the three primary stages of tendon healing: inflammation, proliferation, and remodeling. During the inflammation stage, which lasts up to 48 h, there is an influx of immune cells, including platelets, neutrophils, monocytes, erythrocytes, and macrophages. These cells are guided by a complex interplay of cytokines and growth factors such as Interleukin 6, Interleukin 1-Beta, Fibroblast Growth Factor, Insulin-like Growth Factor, Platelet-Derived Growth Factor, Transforming Growth Factor Beta, and Vascular Endothelial Growth Factor. The proliferation stage, spanning from 3 days to 4 weeks, is characterized by the activation of fibroblasts, macrophages, and tenocytes, along with contributions from Mesenchymal Stem Cells (MSCs) and Tendon-Derived Stem Cells (TDSCs). This stage is critical for the formation of a provisional collagen III (COL3) scaffold, which serves as a template for new tissue. Finally, the remodeling stage, extending from 12 months to up to 2 years, involves the transition from collagen III to the stronger collagen I (COL1), alongside the organization of the extracellular matrix. This phase ensures the strengthening and proper alignment of the collagen fibers, crucial for restoring the tendon’s mechanical properties and function.

Tendon repair is a lengthy process influenced by various factors, including the patient’s overall health and age. This process consists of three phases: inflammation, which occurs shortly after the injury; proliferation, lasting about 3–4 weeks; and remodeling, which may take 1–2 years to complete [3,7,10]. Each phase involves specific cellular activities and biochemical pathways essential for effective healing (Figure 2) [3,7].

Eccentric exercise (EC) can benefit many patients, but it may not be appropriate for everyone due to individual differences in tendon condition, pain tolerance, and other health factors [5,7,11]. Some patients may experience adverse effects or inadequate improvement, requiring alternative or additional therapies. The variability in patient outcomes emphasizes the need for personalized treatment plans in managing degenerative tendinopathy. Patients who do not respond to traditional therapies may receive region-specific treatments, such as extracorporeal shockwave therapy (ESWT) [12].

Additionally, regenerative medicine has shown promise in therapeutics, including blood-derived products like platelet-rich plasma (PRP) and cell-based products such as stem cells (SCs) and stromal vascular fraction (SVF). These products have demonstrated measurable improvements in tissue healing, making them promising therapeutic targets for DT [13].

This review article is critical in the context of the current findings, as it focuses on recent advances in understanding DT and its management. Although much progress has been made in diagnosing DT and understanding its molecular and cellular underpinnings, there remains a significant gap in effective, universally successful treatments. Emerging therapies, such as regenerative medicine, including platelet-rich plasma (PRP) and stem cell-based interventions, show promise but require further exploration to refine their application. This review highlights the need for more personalized approaches that integrate current knowledge to address the variability in patient responses and the limitations of conventional treatments.

### 1.1. Tendinopathy Types and Severity

#### 1.1.1. Rotator Cuff Tendinopathy (RCT)

The rotator cuff consists of four muscles, the supraspinatus, infraspinatus, teres minor, and subscapularis, along with their associated tendons [7]. Rotator cuff tendinopathy (RCT) affects approximately 30% of the general population and accounts for up to 50% of reported shoulder pain cases [7,14]. Supraspinatus tendinitis is the most common type of RCT [14]. The classification of rotator cuff tendinopathy is based on the location and type of the tendon involved. Owing to the complexity of the rotator cuff’s multi-joint system, proper diagnosis and management are essential [15].

Patients with RCT typically experience pain during arm movements, especially when elevating the shoulder or applying a load to the rotator cuff tendons, leading to significant discomfort and functional limitations. Although the exact cause of RCT is unknown, many intrinsic and extrinsic factors are believed to contribute [16]. The microtrauma hypothesis suggests that the repeated overloading of a compromised tendon can result in small tears. If these minor injuries do not heal adequately, continued stress may worsen them, potentially leading to full-thickness tears. The risk of such tears increases with age, highlighting the role of degenerative changes in rotator cuff pathology [17].

Considering both intrinsic factors, such as genetic predispositions and aging, and extrinsic factors, such as occupational hazards and sports participation, is crucial in understanding the onset and progression of tendinopathy as well as the response to various treatments (Table 1) [7,9,13,15].

To identify risk factors for rotator cuff disease, Fabian et al. highlighted four key genes with altered expression profiles: Runt-related transcription factor 2 (RUNX2), Matrix Metalloproteinase 2 (MMP2), collagen 1 alpha 1 (COL1A1), and P2X purinoceptor 7 (P2RX7). These genetic alterations are strongly correlated with the severity of rotator cuff conditions, including the likelihood of full-thickness tears [18]. The roles of these genes suggest their involvement in pathological processes underlying rotator cuff degeneration, such as extracellular matrix remodeling and inflammatory responses [18].

Early diagnosis of RCT is important because pathological changes can lead to tendon rupture. These changes include dysregulation of collagen proteins, such as Collagen 1 (COL1) and Collagen 3 (COL3), collagen matrix degradation, and inflammation [19]. Patients often report pain during internal and external rotations, limiting their activities. As the condition progresses, shoulder movement becomes increasingly restricted. Undiagnosed RCT can lead to long-term complications, including the potential for a rotator cuff tear, which impairs range of motion, strength, and stability, potentially resulting in the development of a long-hooked acromion [20].

Several factors can compromise the post-surgical outcomes of rotator cuff repairs. Jensen and colleagues identified three primary categories influencing the likelihood of retear: (1) the nature of the tear, including its size, extent of fatty degeneration, neuropathy, muscular atrophy, stiffness, degree of retraction, and location; (2) patient health conditions such as diabetes mellitus, hypercholesterolemia, body mass index (BMI), age, use of steroidal anti-inflammatory drugs (NSAIDs), vitamin D deficiency, and osteoporosis; and (3) the type of surgical intervention used and the effectiveness of post-operative rehabilitation [21]. Together, these factors contribute to variability in healing and retear rates [21].

Currently, there is no universally effective treatment for rotator cuff repair, as responses to different therapies can vary significantly among patients. While eccentric exercise (EC) has shown some benefits, and it can reduce pain and improve tendon stiffness and function, thereby gradually restoring normal tendon function, its effectiveness is often limited compared to other therapeutic approaches, because EC may require a longer recovery period, and the initial pain level is higher than other methods [22]. RCTs pose significant challenges, often requiring patients to seek assistance with daily activities. A cross-sectional study revealed that 72% of affected individuals reported poor physical health-related quality of life, and 60% reported poor mental health-related quality of life [23]. This highlights the substantial physical and psychological burden of this condition.

#### 1.1.2. Achilles Tendinopathy (AT)

The Achilles tendon, which is the longest tendon in the human body, consists of fibers from the gastrocnemius and soleus muscles. Achilles tendinopathy (AT) typically results from chronic degeneration and overuse and affects approximately 6% of the general population. It is the most common overuse injury of the ankle and foot, particularly among athletes, accounting for 9% of sports injuries and 18% of running-related injuries [24]. AT is categorized into insertional Achilles tendinopathy (IAT) and mid-portion Achilles tendinopathy (MAT); IAT is diagnosed if the injury is located within 2 cm of the insertion, whereas MAT is diagnosed if the injury is located more than 2 cm from the insertion [25].

Most Achilles tendon problems arise because of overuse and excessive loading (Table 1). Factors contributing to AT include advancing age and, to a lesser extent, inappropriate footwear. Although conventional therapies are available for treating AT, patients who do not respond to traditional treatments may require surgery [26]. However, the mechanisms promoting AT pathophysiology are not fully understood. Researchers also identified the factors that may contribute to AT, including altered homeostasis, mechanical overloading, fibrosis, hypoxia, and upregulation of the glutamate NMDA receptor subunit 1 (NMDAR1) [27,28]. Pathogenesis of AT is characterized by collagen disruption, which is followed by increased levels of collagen type III (COL3), increased vascularity, and reduced levels of collagen type I (COL1). This insufficient healing process leads to a cascade of dysregulation, which results in the degeneration of the tendon [29].

Approximately one-third of AT cases involve IAT, a particularly painful subtype that often responds poorly to non-surgical treatments. While slow eccentric loading exercises may provide symptom relief for some patients, a significant number ultimately require surgical intervention. The exact pathophysiology of IAT remains incompletely understood. However, research suggests that IAT is characterized by significant alterations in the tendon’s compressive mechanical properties and increased calcium deposition. These morphological changes are associated with a progressive decline in tendon quality and function [30].

Early detection and prediction of Achilles tendinopathy (AT) remain challenging due to the absence of definitive biomarkers. Nevertheless, some studies have reported that a lower shear modulus in tendons is associated with improved clinical outcomes, with significant progress observed up to six months post-treatment. Conversely, a higher shear modulus has been correlated with worse patient-reported symptoms; other research has underscored the role of growth factors in tendon recovery, particularly fibroblast growth factor (FGF), which has been linked to higher Achilles tendon rupture scores (ATRSs) and enhanced collagen synthesis, as evidenced by elevated COL1 levels [31,32]. Despite the availability of numerous treatment modalities, inconsistent clinical data continue to impede the development of a standardized and effective treatment strategy for AT [33].

#### 1.1.3. Patellar Tendinopathy (PT)/Jumper’s Knee

Patellar tendinopathy (PT), commonly known as ‘Jumper’s Knee’, occurs when the patellar tendon, which connects the patella to the tibia and aids in knee extension and alignment, experiences accumulated stress, which leads to microtears. This condition is prevalent among athletes involved in sports that require repetitive and intense loading of the patellar tendon, such as volleyball and basketball, affecting 30–50% of players [34,35,36]. While most PT cases respond well to non-operative treatments, surgical intervention may be necessary if conservative therapies do not provide relief after six months [37] (Table 1).

PT pathology involves changes in the tendon and extracellular matrix (ECM). Studies have reported significant morphological and elastic alterations in individuals with unilateral patellar tendinopathy (UPT). Patients with UPT demonstrated increased tendon thickness and stiffness, as well as a higher shear modulus, when compared to asymptomatic individuals. These biomechanical changes persisted for up to six years, suggesting long-term structural and functional adaptations associated with the condition. [38]. Dysregulation of the extracellular matrix (ECM) plays a critical role in the pathogenesis of patellar tendinopathy (PT). Studies have reported degenerative alterations in the ECM of the patellar tendon, notably with a significantly elevated deposition of sulfated glycosaminoglycans in pathological tendons compared to healthy controls [39]. In another study, researchers observed elevated levels of proteoglycans (PGs) and versican within the ECM of tendinopathic tendons, with PG levels being 25-fold higher in PT tendons than in healthy controls. Excess PGs may contribute to altered ECM homeostasis, resulting in abnormal tendon structure and composition [40].

Risk factors for PT include various anthropometric differences, such as body mass index (BMI), body weight, waist–hip ratio, and leg-length discrepancies. Other factors include increased tendon load, poor range of motion, and decreased strength and flexibility of the quadriceps and hamstrings [41]. These factors can predispose individuals to tendon stress and subsequent injury.

Patellar tendinopathy (PT) is categorized into several stages to guide the selection of appropriate therapeutic interventions. In the initial stage, patients experience pain in the patellar tendon during physical activity, which may fluctuate intermittently. If left untreated, this pain can persist even at rest and may eventually lead to patellar tendon rupture [42]. Non-surgical treatment modalities are effective for most patients and include orthobiologic therapies such as platelet-rich plasma (PRP), stromal vascular fraction (SVF), and stem cells, as well as eccentric exercise (EC), extracorporeal shockwave therapy (ESWT), and nutritional management. For individuals who do not respond adequately to these conservative treatments, surgical intervention is indicated. However, recurrent pain and re-rupture have been reported in some cases post-surgery, limiting further therapeutic options. For patients with persistent symptoms following failed surgical intervention, multiple longitudinal tenotomies have demonstrated significant clinical improvements, as evidenced by a 30-month follow-up study [43].

In most patients, proper rehabilitation and management yields positive clinical outcomes. This generally involves avoiding tendon overuse and excessive loading during recovery. Physicians often design detailed eccentric training programs to aid rehabilitation, as eccentric training has been shown to improve tendon function and muscle strength [44]. Eccentric exercises have been reported to facilitate the breakdown of damaged or degenerative tendon tissue, which is subsequently replaced by newly synthesized, healthier collagen. This remodeling process plays a critical role in restoring long-term tendon health and function [45]. However, despite the well-documented benefits of eccentric loading, a subset of patients may fail to respond to this approach alone. For these individuals, the development of novel, non-surgical interventions and personalized rehabilitation protocols is essential to achieve optimal therapeutic outcomes.

#### 1.1.4. Lateral Elbow Tendinopathy/Tennis Elbow/Lateral Epicondylitis (LET)

Overuse of the extensor carpi radialis brevis (ECRB) tendon can lead to microinjuries, resulting in lateral epicondylitis (LET), commonly referred to as tennis elbow. This condition is frequently reported in tennis players, with approximately 75% of them experiencing tennis elbow pain due to constant grip and wrist extension. LET is characterized by persistent elbow pain. Fortunately, the prognosis of LET is generally good, as the majority of patients report spontaneous recovery. Initial treatment typically includes conservative measures, such as applying ice, rest, and non-steroidal anti-inflammatory drugs (NSAIDs), for pain relief. Physical and occupational therapy is recommended before considering surgical options [46].

The exact pathogenesis of lateral epicondylitis (LET) remains unclear, though certain genetic factors have been identified as potential risk contributors. Recent investigations have focused on the expression profiles of two key neuromediators—Substance P (SP) and calcitonin gene-related peptide (CGRP)—both of which are implicated in LET. These mediators are expressed in the extensor carpi radialis brevis (ECRB) tendon, where they are thought to trigger an inflammatory response following tendon injury. Elevated levels of SP and CGRP have been detected in tendon samples from patients with LET, with their expression strongly correlating with the extent of tendon degeneration [47]. Additionally, structural abnormalities in the tendons of individuals engaged in sports involving repetitive loading of the upper limbs have been reported. Through single nucleotide polymorphism (SNP) genotyping, a significant association between the COL11A1 rs3753841 variant and LET pathology was identified, further supporting the notion that genetic predisposition plays a role in tendinopathy risk [48].

Despite LET being relatively straightforward to manage, rest is frequently recommended to prevent symptom exacerbation. If pain persists, physiotherapy is the preferred treatment approach, as rehabilitation interventions generally offer superior outcomes in terms of functional improvement and pain relief compared to non-steroidal anti-inflammatory drugs (NSAIDs) or corticosteroids. However, if symptoms fail to improve after 12 months of conservative treatment, NSAIDs may be considered, and in severe cases, surgical intervention may be necessary [49] (Table 1).

**Table 1:** Characteristics of tendinopathy types.

Tendinopathy Types	Symptoms	Reported Causes	Treatment
Rotator Cuff tendinopathy (RCT)	Patients with RCT commonly experience pain during shoulder movement and elevation [14,15]. They also report restricted range of motion, particularly during internal and external rotations [20].	RCT is primarily caused by overloading of the rotator cuff tendons. Additionally, dysregulation of various intrinsic and extrinsic factors contributes to the condition [15,16,20]. Altered expression of genes such as RUNX2, MMP2, COL1A1, and P2RX7 has been linked to the severity of rotator cuff pathology [18].	Treatment decisions are based on multiple factors, including the extent of the tear and patient comorbidities like diabetes, BMI, and age [21]. Eccentric training is typically recommended, though some patients may respond to NSAIDs, while others may require surgical intervention [20,21,22].
Achilles Tendinopathy (AT)	AT presents with pain, swelling, and stiffness of the Achilles tendon. Patients often display impaired biomechanical properties of the tendon, alongside increased calcium deposits [30].	Similar to RCT, AT arises from various factors, including mechanical overloading, aging, elevated NMDAR1 levels, and disrupted collagen homeostasis [27,28].	Initial treatment generally involves rest, followed by physical and occupational therapy [30,46]. Surgical intervention is considered for patients who do not respond to conservative treatments [30].
Patellar Tendinopathy (PT)/Jumpers Knee	PT causes intense anterior knee pain, particularly localized to the patella’s inferior pole [37].	The primary cause of PT is accumulated stress on the patella, leading to microtears. Repetitive loading and motion further exacerbate the condition [35,36].	Initial management involves physical rehabilitation. In cases where conventional treatment fails, orthobiologics like platelet-rich plasma or stromal vascular fraction may be recommended [42]. Surgery is reserved for patients who do not find relief with these treatments [43].
Lateral Elbow Tendinopathy/Tennis Elbow/Lateral epicondylitis (LET)	LET manifests as persistent pain localized to the lateral aspect of the elbow [46].	Overuse and repetitive strain of the extensor carpi radialis brevis (ECRB) muscle are common causes of LET [46]. Genetic predispositions, such as variations in COL5A1 and COL11A1, have also been identified as risk factors [48].	LET is typically managed through rest and avoidance of activities that worsen symptoms [46]. Surgery is only considered if conservative treatments fail to provide symptomatic relief [49].

### 1.2. Molecular Changes in Extracellular Matrix

The extracellular matrix (ECM) is an integral part of tendons. Appropriate composition and organization of the ECM help the tendon perform its mechanical function and ensure the biological function of the tendon. The ECM of healthy tendons is predominantly composed of collagen (COL). COL1 constitutes 90% of the tendon ECM, while the remainder consists of COL III, V, XI, XII, and XIV. In addition to collagen, there are a variety of other non-collagenous matrix components. They are divided into proteoglycans, glycoproteins, and glycoconjugates [50].

The tendon cell population predominantly comprises tenocytes. Because no tendon-specific markers exist, the following markers are known to be highly expressed by tenocytes: Scleraxis (SCX), Tenomodulin (TNMD), Mohawk x (MKx), and Tenascin C (TNC). Additionally, a resident stem cell population known to exist within the tendon is called tendon-derived stem cells (TDSCs). These TDSCs have shown characteristics of stem cells, including multipotency and self-renewal capacity [51].

Tendon pathology is accompanied by several molecular changes. An important class of enzymes called matrix metalloproteases (MMPs) is involved in the development of tendinopathy. MMPs can degrade the tendon ECM, which is a hallmark of tendinopathy. To counterbalance the activity of MMPs, another class of enzymes called tissue inhibitors of metalloproteases (TIMPs) exists. The altered expression of MMPs and TIMPs can disturb tendon homeostasis, which may play a role in the pathogenesis of tendinopathy [52]. Previously, it was reported that MMPs, in part, may promote the pathogenesis of tendinopathy [5]. Therefore, a balance between TIMPs and MMPs is necessary for tendon homeostasis [53].

Aging is a critical factor that induces various physiological changes, including disruptions in the homeostatic regulation of matrix metalloproteinases (MMPs) and tissue inhibitors of metalloproteinases (TIMPs) [52,53,54]. Extensive research has highlighted the role of MMPs and TIMPs in the development of chronic tendon pathologies. For example, a study by Yu et al. investigated the effects of aging on the activity of MMPs and TIMPs, revealing a significant upregulation of MMP-2 and MMP-9 levels in aging tenocytes compared to TIMP-1 and TIMP-2. This imbalance suggests that aging may compromise tendon structure and impair healing processes by promoting an overactive MMP-mediated matrix degradation. Such findings underscore the need for further investigation into therapeutic strategies aimed at restoring the MMP/TIMP equilibrium to preserve tendon health in aging populations [53,55]. In addition to maintaining the balance between matrix metalloproteinases (MMPs) and tissue inhibitors of metalloproteinases (TIMPs), several therapeutic strategies have been proposed to support extracellular matrix (ECM) recovery following tendon injury. One such approach is targeted exercise, which enhances the tendon’s ability to tolerate increased mechanical loads, improves its mechanical properties, and stimulates anabolic processes, thereby facilitating ECM homeostasis. Furthermore, orthobiologics like platelet-rich plasma (PRP) have demonstrated potential in promoting stem cell differentiation into tenocytes, increasing collagen synthesis, and enhancing cellular proliferative activity [52,54,56]. This approach increases the cell population that can respond to injury and aids in matrix remodeling [54,57].

### 1.3. Cellular Changes in the Pathogenesis of Tendinopathy

Tendinopathy is characterized by distinctive histopathological, clinical, and radiological findings. Histopathologically, tendinopathy involves collagen disorganization, increased deposition of mucoid ground substance, heightened cellularity, and the presence of rounded, plump “chondroid” cells indicative of the ossification process often seen in tendinopathy [58]. As tendon degeneration progresses, the markers of healing diminish, resulting in a pathology akin to chondroplasia, where normal tendon architecture is replaced by fibrocartilaginous tissue exhibiting hyaline, mucoid, calcified, or fibrous characteristics [59].

Beyond structural alterations, inflammation is a key contributor to tendinopathy’s pathogenesis and progression, particularly in its early stages, where clinical symptoms may remain undetectable despite clear histological evidence of tissue damage. Recent studies have identified various inflammatory mediators and immune cell populations, such as macrophages, T cells, and mast cells, as pivotal drivers of the underlying pathology in tendinopathy [60]. Emerging data indicate that alarmins, released from necrotic cells, initiate an inflammatory cascade that activates the innate immune system [61]. These inflammatory changes induce cellular alterations, including modifications in cell shape, and are associated with elevated levels of pro-inflammatory mediators and extracellular matrix degradation [62].

Despite these pathological challenges that impair tendon function and regeneration, considerable advancements have been made in developing therapeutic interventions to promote tendon healing and repair. These strategies encompass pharmacological therapies, targeted physical rehabilitation programs, and innovative regenerative approaches. A detailed discussion of these therapeutic modalities and their clinical efficacy will be presented in the subsequent section.

### 1.4. Treatment Options

Over the years, a variety of treatment strategies have been developed, each with differing levels of effectiveness. This section will explore these options, including eccentric training, dietary and nutritional interventions, extracorporeal shock wave therapy (ESWT), anti-inflammatory treatments, platelet-rich plasma (PRP) and PRP-derived growth factors, adipose-derived stromal vascular fraction (ADSVF/SVF), hormonal regulation, stem cell therapy, and epigenetic regulators, alongside their reported clinical outcomes.

#### 1.4.1. Eccentric Training (EC)

EC is considered the first-line treatment for tendinopathies [63]. For instance, both eccentric and aerobic exercises have been found to improve function in patients with upper limb tendinopathies [63,64,65]. Furthermore, EC was found to improve functionality in patients with upper limb tendinopathy [63,66,67]. EC treatment has been associated with molecular changes, such as promoting the synthesis of type 1 collagen in patients with Achilles tendinosis (ATS) where COL1 expression levels were significantly higher than in controls [67,68]. Another study reported that EC increased serum levels of Insulin-Like Growth Factor 1 (IGF-1), which is involved in tendon healing and cellular proliferation, making it a therapeutic target for tendinopathy [68,69]. Vascular Endothelial Growth Factor (VEGF) is another critical gene whose dysfunction is well documented in tendinopathy. VEGF plays a critical role in angiogenesis and tendon healing, and following exercise, VEGF levels are elevated in both sedentary and athletic individuals. This suggests that enhanced angiogenesis likely contributes to tendon repair [70].

From a clinical perspective, the EC programs for tendinopathy rehabilitation frequently incorporate modified versions of the Alfredson or Silbernagel protocols. In one study, clinicians utilized a modified Alfredson protocol and reported a significant reduction in pain scores among patients with chronic tendinopathy [71]. Similarly, the Silbernagel protocol, a 12-week regimen emphasizing controlled overload through eccentric training, produced favorable clinical outcomes, including improved range of motion in all participants [72]. A comparative study found no significant differences in clinical outcomes between the Alfredson and Silbernagel protocols, indicating that both are effective when implemented under supervised guidance for tendinopathy management [73,74]. Overall, eccentric training is a safe and efficacious intervention, demonstrating superior improvements in strength and flexibility compared to other strength training methods [74].

Wernbom et al. highlighted that delayed-onset muscle soreness (DOMS) is a well-documented adverse effect of eccentric loading, which can be mitigated through a gradual, graded exposure to eccentric muscle contractions [75]. However, in clinical populations, a potentially more critical concern with isolated eccentric contractions is the issue of training specificity. This poses a particular challenge for patients with impaired concentric strength, as it may hinder the effective transfer of eccentric gains to concentric strength improvements, thereby limiting clinical outcomes and prolonging dysfunction and pain [76]. Additionally, a study by Rikke Beyer et al. emphasized that patient adherence to prescribed eccentric exercise protocols plays a crucial role in determining treatment prognosis [77]. These factors collectively restrict the broader clinical applicability of eccentric contractions to some extent.

#### 1.4.2. Diet and Nutrition

In addition to physical interventions, diet and nutrition are crucial for tendon recovery and prevention. For example, vitamin C (VC) has garnered attention because of its antioxidant properties and role in collagen synthesis. A recent meta-analysis indicated that VC supplementation can promote collagen production, thereby aiding tendon repair. Conversely, VC deficiency has been linked to decreased pro-collagen production, hindering the healing process [78]. VC-enriched gelatin has also been shown to upregulate collagen synthesis, with effects lasting up to 72 h post-consumption, highlighting VC’s potential to enhance tendon health and metabolism [79].

Furthermore, the efficacy of VC is enhanced when used in combinations with other nutraceuticals. A study involving a combination of type 1 collagen peptide, chondroitin sulfate, sodium hyaluronate, and VC reported significant improvements in tendon repair and pain reduction compared with a control group [80]. This underscores the importance of exploring various bioactive compounds for tendon therapies.

Curcumin, a well-known anti-inflammatory dietary supplement, has been investigated in recent studies for its potential therapeutic effects on tendon injuries. In a rat model of Achilles tendon (AT) injury, curcumin delivered via porous microspheres was shown to suppress pro-inflammatory cytokines in a dose-dependent manner, highlighting its potential role in tendon healing and repair [81]. Furthermore, a combination of curcumin and Boswellia-extract significantly alleviated pain and improved functional outcomes in patients with chronic and early-stage tendinopathy, presenting a promising alternative to non-steroidal anti-inflammatory drugs (NSAIDs) with fewer adverse effects [82]. These findings suggest that nutraceuticals could offer a valuable approach to tendinopathy management. However, further research is needed to clarify their mechanisms of action and to optimize their application in clinical practice. The nutraceuticals such as curcumin and Boswellia show potential as treatments for tendinopathy, but additional studies are required to fully understand their therapeutic mechanisms and clinical efficacy.

#### 1.4.3. Extracorporeal Shock Wave Therapy (ESWT)

Extracorporeal shock wave therapy (ESWT) is increasingly recognized as a safe and efficacious intervention for various tendinopathies, including plantar fasciitis (PF), lateral elbow tendinopathy (LET), and patellar tendinopathy (PT). It can be administered using either high-energy or low-energy shock waves, and evidence suggests that high-energy ESWT yields superior functional and imaging outcomes. The precise mechanisms underlying the efficacy of ESWT remain incompletely elucidated, but ESWT is hypothesized to alleviate pain through multiple pathways, including analgesic effects that modulate the serotonergic system responsible for pain regulation and hyperstimulation of affected tissues [83].

Emerging hypotheses suggest that ESWT may activate pro-inflammatory and catabolic processes, facilitating the clearance of damaged extracellular matrix components [84]. Additionally, ESWT is postulated to stimulate tenocyte proliferation and collagen synthesis, both of which are critical for tendon repair [85,86]. The mechanical impact of shock waves may also induce microdisruptions in avascular or poorly vascularized tissues, triggering neovascularization, enhancing the blood supply, and promoting tissue regeneration [87,88].

Despite these benefits, the efficacy of ESWT for tendinopathy remains a subject of debate. The majority of randomized controlled trials (RCTs) reported significant improvements in pain reduction, functional recovery, and tissue repair following ESWT [89]. In particular, a study with 60 participants with Achilles tendinopathy (AT) demonstrated that ESWT significantly alleviates symptoms in both insertional (IAT) and non-insertional Achilles tendinopathy (NIAT), with IAT patients exhibiting superior clinical outcomes [90,91]. Similarly, a recent meta-analysis of clinical trials reported a substantial reduction in pain associated with ESWT across various tendinopathy types [91]. Furthermore, combining ESWT with eccentric exercises and stretching has demonstrated superior outcomes compared with ESWT alone in mid-portion Achilles tendinopathy [24].

Nevertheless, several studies have reported conflicting results [89]. For instance, a systematic review and meta-analysis concluded that ESWT did not provide significant improvements in pain or function compared with controls, although this analysis only included studies published up to 2013, potentially limiting its contemporary relevance [92].

Recent evidence suggests that low-energy ESWT may offer therapeutic benefits comparable to those of high-energy protocols, potentially inducing molecular changes that promote tendon repair. Studies have demonstrated that low-energy ESWT can stimulate the expression of Vascular Endothelial Growth Factor (VEGF) in endothelial cells, reduce cell death, and promote vascularization [93]. The combination of ESWT with platelet-rich plasma (PRP) injections has been shown to enhance growth factor activity at the injury site, facilitating tendon regeneration [94]. Growth factors such as VEGF are particularly important for vascularization and tissue healing, offering a promising non-surgical treatment option for severe musculoskeletal disorders [95]. Therefore, ESWT is widely regarded as a safe and efficacious treatment for tendinopathies. However, further large-scale studies are necessary to elucidate its mechanisms and optimize its clinical application.

#### 1.4.4. Anti-Inflammatory Treatment

Anti-inflammatory treatments, such as NSAIDs and glucocorticoids (GCs), are commonly used for pain relief in tendinopathies. NSAIDs are known for their short-term analgesic benefits in certain patients; however, the long-term use of NSAIDs has been reported to cause severe side effects, including negative effects on the gastrointestinal tract, cardiovascular system, kidneys, and liver. Additional evidence suggests that NSAIDs are ineffective for treating tendon injuries and are associated with an increased risk of adverse effects. Importantly, the healing process of tendinopathy does not appear to be influenced by NSAID consumption [96].

However, it has been shown that even short-term beneficial effects of NSAIDs may be patient specific. Heinemeier et al. observed that one-week ibuprofen treatment in individuals with Achilles tendinopathy did not initiate a reparative response in the affected tendon tissue, and any potential pain relief was moderate [97].

Long-term NSAID use has been reported to potentially impair bone and tendon functions [96]; thus, their use in tendinopathy remains controversial owing to limited efficacy in promoting tendon healing [98]. For example, Malmgaard et al. found no short- or long-term benefits of NSAIDs in combination with physical rehabilitation, reporting no changes in tendon thickness or improved neovascularization [98]. Moreover, the analgesic properties of NSAIDs may lead athletes to apply excessive stress on their tendons prematurely, undermining long-term recovery [96].

The mechanism by which NSAIDs may induce detrimental effects has been demonstrated by Bittermann et al. Their findings concluded that NSAID use during the inflammatory phase of tendinopathy may disrupt extracellular matrix remodeling and impair the regulation of inflammatory and wound-healing genes. NSAID-mediated COX2 inhibition likely hinders the turnover of a pro-inflammatory hyaluronic acid matrix, delaying the transition to cellular processes essential for functional matrix remodeling and healing [99].

Similarly, despite their strong anti-inflammatory properties, GCs offer limited benefits, which are primarily confined to short-term pain relief. A meta-analysis suggested that overuse of GCs may lead to detrimental effects, necessitating moderate dosing and careful monitoring to prevent complications [100,101]. Proper rehabilitation and post-treatment management are crucial for the success of GC therapy. Johannsen et al. found that combining GC with exercise significantly improved patient outcomes compared with exercise alone [102].

Both selective COX-2 inhibitors and non-selective NSAIDs have been shown to provide short-term pain relief for rotator cuff tendinopathy. However, caution is warranted in the use of COX-2 selective NSAIDs, as they are associated with higher retear rates compared to traditional non-selective NSAIDs. [103]. Despite offering similar efficacy to corticosteroid injections, the long-term effects of COX-2 inhibitors on pain, tendon healing, and overall function remain a subject of concern and warrant further investigation [104]. Although NSAIDs have been a mainstay in managing tendinopathies, emerging evidence suggests that alternative treatments—both pharmacological and non-pharmacological—may offer superior therapeutic benefits. Eccentric training has proven effective in strengthening the affected tendon and enhancing flexibility [105], while extracorporeal shock wave therapy (ESWT) has shown potential in stimulating tendon tissue repair and reducing pain [106].

Dry needling is another technique reported to alleviate pain and improve tendon function [107]. Additionally, advanced biological therapies, such as platelet-rich plasma (PRP) and stem cell injections, offer promising regenerative effects [108,109]. PRP promotes healing through the release of growth factors from concentrated platelets, while stem cells facilitate tissue repair and regeneration by directly stimulating cellular processes. Future research should focus on exploring the synergistic potential of combining glucocorticoids (GCs) and NSAIDs with these evolving therapeutic modalities. Clarifying the long-term impacts of these combined approaches on tendon repair, function, and pain management will be crucial to optimizing treatment outcomes for rotator cuff tendinopathy

### 1.5. Advancements in Therapeutic Approaches

NSAIDs, corticosteroids, extracorporeal shock wave therapy (ESWT), and physical rehabilitation can offer some relief, but they often do not provide consistent or long-term results and have limitations. New therapies are being developed to overcome these issues using biological and minimally invasive methods [7]. These include orthobiologics such as platelet-rich plasma (PRP), stromal vascular fraction (SVF), and various types of stem cells [1,4,7,24,95,110,111]. While these therapies have shown positive clinical outcomes in both the short and long term, as indicated in Table 2, they can have minor complications that need to be addressed. Additionally, their use in clinical settings is limited owing to specific constraints, which are discussed in this article.

In addition to these therapies, new, minimally invasive surgical techniques have been developed. For example, endoscopy and electrocoagulation focus on disrupting abnormal neoinnervation rather than directly addressing the lesion. These procedures offer alternative solutions in cases that are unresponsive to conservative treatments [7,16,42,43]. The exploration of emerging therapies highlights the need for consistent long-term treatment of tendinopathy. Further research and clinical trials are essential to confirm the safety and efficacy of these biologics, which show promise in restoring tendon properties and reducing the risk of re-injury.

### 1.6. Biologics

Biologics are emerging as potential treatments for tendinopathy, a condition characterized by pain and tendon dysfunction. Various biologics, PRP, and stem cell therapies, have been explored for their potential to facilitate tendon healing [7,10,13].

#### 1.6.1. Platelet Rich Plasma (PRP) and PRP-Derived Growth Factors

Platelets are the primary component of platelet-rich plasma (PRP) and contain over 1500 bioactive factors, including growth factors, enzymes, and immune messengers. Thus, PRP is an ideal therapeutic option in regenerative medicine because of its potential to accelerate the healing of soft tissue, cartilage, and bone. Therefore, this is a safe and minimally invasive treatment option. Numerous in vivo studies have demonstrated PRP’s effectiveness in promoting tendon healing, showing significant improvements in the treatment of tendinopathy [111,136,137].

PRP preparation involves the use of commercially available kits, and a standardized dose is administered for all types of tendon injuries, regardless of the patient’s age, sex, or medical history. Key factors in PRP preparation include centrifuge parameters, blood drawing techniques, platelet content, and activation [138].

PRP aids in tendon repair by reducing inflammation and pain while enhancing cellular proliferation. In vitro studies have indicated that PRP upregulates the expression of collagen type I (COL1), collagen type III (COL3), and other tendon-related genes and proteins [138,139]. Additionally, various growth and angiogenic factors present in PRP, such as Epidermal Growth Factor (EGF), Insulin-like Growth Factor 1 (IGF-1), Hepatocyte Growth Factor (HGF), Transforming Growth Factor beta (TGF-β), Vascular Endothelial Growth Factor (VEGF), Platelet-derived Growth Factor (PDGF), and Fibroblast Growth Factor (FGF), significantly contribute to tissue repair. For instance, FGF in PRP promotes fibroblast formation and COL1 synthesis, which enhances neovascularization and bone healing [140].

PDGF and TGF-β are involved in the regulation of cellular proliferation and play roles in collagen synthesis during development and adulthood. At the wound site, PDGF can promote collagen deposition, crosslinking, and the biomechanical properties of healing tendons. Additionally, it may increase vascularization and further support tendon repair [141]. PDGF plays a critical role in the early stages of tendon healing as it mediates the early inflammatory response by recruiting and activating wound macrophages. M1 macrophages are essential for extracellular matrix (ECM) deposition, while M2 macrophages release growth factors (including TGF-β, VEGF, and IGF-1) to stimulate tissue repair. Furthermore, M2 macrophages can promote angiogenesis by directly regulating the proliferation of vascular endothelial cells and inducing cell migration through the PDGF receptor-β (PDGFR-β). Additionally, PDGF-BB stimulates angiogenesis by inducing erythropoietin production in stromal cells [142].

VEGF is a crucial growth factor known to enhance blood flow to tendons and ligaments. Increased VEGF levels in damaged tissues correlate with greater vascularization. VEGF is secreted by platelets at injury sites and plays a critical role in promoting angiogenesis during the proliferative phase of healing. Specifically, it increases vascular wall permeability and stimulates the growth of both vascular endothelial and perivascular cells [143]. In a study by Shams and co-workers, VEGF enhances fibroblast proliferation, improve tissue regeneration, and promotes wound repair in vivo [144]. PRP treatment was shown to promote VEGF expression, thereby enhancing neovascularization in rat models. This finding highlights the importance of PRP-derived VEGF in accelerating the bone-healing process [140]. Although the exact mechanisms are not fully understood, VEGF may exert beneficial effects via several pathways. One proposed mechanism through which VEGF may promote tendon repair is by inhibiting adipogenic differentiation of tendon-derived stem cells (TDSCs) by targeting secreted phosphoprotein 1 (SPP-1), thereby facilitating their differentiation into tenocytes [145]. Additionally, VEGF treatment may boost the healing mechanism by promoting tenocytic proliferation and downregulating cellular apoptosis. This was followed by enhanced production of COL1, inhibition of COL3, and reduced ECM degradation as a result of TIMP upregulation and MMP downregulation. These molecular processes convert a typically prolonged inactive early-to-middle healing phase into a more biologically active phase, resulting in significant improvement in tendon biomechanical properties [146].

Another important component of PRP, IGF-1, is known to exert anabolic effects by enhancing deoxyribonucleic acid (DNA) content and promoting collagen synthesis. It plays a crucial role in adult tendon growth in response to mechanical loading [147]. In addition, IGF-1 does not function by decreasing the migration of inflammatory cells. Instead, it appears to inhibit the early stages of the inflammatory cascade through a negative feedback mechanism that leads to an increase in the IGF-1 concentration [148]. Disser et al. investigated the role of IGF-1 in adult tendons and found that it promotes the expression of early growth response genes Egr1 and Egr2, as well as the transcription factor Scleraxis (SCX), which are essential for tendon development. They also demonstrated that IGF-1 induces Snai1 expression, which is a key factor in fibroblast-mediated tissue growth. Additionally, the study revealed that IGF-1 activates the PI3K/Akt and ERK signaling pathways, underscoring its role in tenocytic proliferation and protein synthesis [149].

In vivo studies demonstrated the therapeutic potentials of PRP. In a rat model of Achilles tendinitis (AT), a single PRP injection significantly increased COL1 levels and improved tendon repair compared to the control group [141]. It was also reported that a combination of PRP and TDSCs enhanced tendon healing more effectively than PRP alone, as evidenced by the increased expression of tenocyte-related markers [150].

Clinical studies have also highlighted the benefits of PRP in tendinopathy treatment. Wesner and co-workers reported that patients with chronic rotator cuff tendinopathy experienced significant pain reduction and functional improvement after PRP injections [150]. Similarly, Mazzocca and co-workers reported that PRP injections were effective in reducing pain and improving function in patients with chronic elbow tendinopathy [151]. However, during a 10-year follow-up, researchers found that clinical and radiographic results showed that the results obtained with and without PRP injections were significantly different. Small differences observed in the 2-year follow-up disappeared in the long term. Patient satisfaction remains high for 10 years after surgical treatment [152]. The use of PRP to augment arthroscopic rotator cuff repair remains an interesting but controversial option. Despite the promising outcomes associated with PRP, standardized protocols regarding PRP preparation, dosage, and application methods are still required. Additionally, more extensive clinical trials are required to establish the most effective PRP treatment strategy for various tendon injuries [153].

#### 1.6.2. Adipose-Derived Stromal Vascular Fraction (ADSVFS, SVF)

The stromal vascular fraction (SVF) is a heterogeneous population of cells isolated from adipose tissue. It is separated using collagenase and comprises mesenchymal stem cells (MSCs), smooth muscle cells (SMCs), macrophages, and endothelial precursor cells (EPCs). The use of SVF in regenerative medicine has garnered attention owing to its role in promoting wound healing, bone repair and regeneration, and enhanced cardiac function, among other applications [154].

The efficacy of stromal vascular fraction (SVF) in tendon repair has been investigated in various preclinical and clinical models. In a rabbit model of flexor tendon injury, SVF treatment significantly enhanced tendon repair, evidenced by increased collagen type I (COL1) expression and improved fibrillar linearity post-surgery [155].

Notably, a marked reduction in vascularity in the treated tendons suggested accelerated remodeling and healing compared to controls. In the animal model of tendinopathy, the combination of SVF with adipose micro-grafts (AMGs) showed further potential for tendon regeneration. This approach was hypothesized to exert anti-inflammatory effects and to upregulate the expression of Factor VIII-related antigen (FVIIIRa), a key mediator of angiogenesis. The treatment improved tendon thickness and stiffness, restoring biomechanical properties to levels comparable to those of healthy tendons. Given its safety profile and therapeutic efficacy, SVF is being considered as a viable alternative to traditional anti-inflammatory medications in managing tendon pathology. A marked reduction in vascularity was also observed, indicating accelerated tissue remodeling and maturation following SVF administration. In another study utilizing an ovine model of tendinopathy, the combination of SVF with adipose micro-grafts (AMGs) demonstrated notable therapeutic effects. This combination not only induced an anti-inflammatory response but also upregulated Factor VIII-related antigen (FVIIIRa) expression, which is crucial in angiogenesis. This dual effect contributed to improved tendon thickness and stiffness, restoring characteristics comparable to normal tendon tissue. Given these outcomes, SVF has been suggested as a viable alternative to conventional anti-inflammatory therapies, presenting a promising avenue for tendinopathy management [156].

Clinicians have also reported the potential benefits of SVF. In a case involving a professional athlete with Achilles tendinopathy (AT), SVF treatment led to significant functional improvements within one month. Although MRI assessments revealed no detectable changes in lesion characteristics, these findings suggest that SVF may promote tendon healing through underlying mechanisms that remain to be elucidated [132].

Furthermore, in a study on the efficacy of SVF versus platelet-rich plasma (PRP) for AT treatment, both interventions were found to be safe and effective in alleviating pain and enhancing functionality. However, SVF demonstrated superior outcomes, with significant improvements observed as early as 15 days post-treatment, making it a potentially more suitable option for patients requiring rapid recovery [125].

Long-term outcomes have also been reported. A 5-year follow-up study reported significantly better visual analog scale (VAS) and Western Ontario and McMaster Universities Arthritis Index (WOMAC) scores in patients receiving SVF compared to those treated with hyaluronic acid (HA), suggesting a sustained therapeutic effect of SVF in musculoskeletal disorders [157]. Although SVF is generally well-tolerated, some adverse events, such as arterial hypertension, chest pain, and transient knee swelling, have been documented in isolated cases [158]. Santoprete et al. observed knee swelling in 7% of patients, which was transient and self-limiting [159].

These adverse events may be influenced by patient-specific factors and warrant further investigation.

Despite its therapeutic promise, certain limitations exist in the application of SVF. Being a heterogeneous cell mixture, the efficacy of individual cellular components within SVF has yet to be fully characterized. Determining the optimal cell concentration and injection environment is critical for achieving the best therapeutic outcomes. Furthermore, the anatomical site of fat collection and patient age can affect the yield and quality of SVF-derived cells, highlighting the need for standardized protocols and possibly combining SVF with other biologics to expand its clinical applicability [160,161,162]. While SVF presents a compelling alternative in tendon repair, further research is necessary to refine its use and fully unlock its regenerative potential.

### 1.7. Diet Supplements

The use of dietary supplements for the treatment of tendon injuries is still being explored. Previous studies have highlighted the importance of nutritional and metabolic factors for tendon healing. Dietary supplements, including collagen-derived peptides (CDP) and vitamin C (VC), have shown potential in reducing tendon pain.

Bollus et al. evaluated the efficacy of combined mucopolysaccharide (COL1) and VC (MCVC) supplementation. The MCVC was used in conjunction with an eccentric (EC) training program for the treatment of Achilles tendinopathy (AT). This combinatorial treatment was more effective in reducing pain associated with reactive tendinopathy than EC training alone [163].

A bioengineered soft tissue repair matrix (STRM), composed of human type I collagen (COL1) and autologous platelet-rich plasma (PRP), has been utilized for the treatment of lateral epicondylitis (LET), demonstrating promising clinical outcomes. Patients experienced significant functional improvement, with observed structural tendon repair. Importantly, no adverse effects were reported during short-term follow-up, highlighting the matrix’s potential therapeutic value. However, the long-term efficacy and safety of STRM require further investigation to confirm its sustained benefits [164].

Ultrasound-guided CDP injection was also used as a treatment for partial-thickness rotator cuff tear. Additionally, ultrasound-guided collagen-derived protein (CDP) injections have been employed in the management of partial-thickness rotator cuff tears (RCTs). In conjunction with kinesiotherapy, patients were assessed both before and after treatment, revealing marked clinical improvement by the final follow-up. Ultrasound imaging further supported these outcomes, showing progressive healing of the rotator cuff tear and enhanced tendon repair, suggesting the potential of CDP as a viable therapeutic option for tendon regeneration in RCT cases. Further studies are needed to fully validate the efficacy and long-term benefits of this approach [165].

### 1.8. Hormonal Regulation

Hormonal imbalance may play a significant role in tendon disorders. It has been reported that individuals suffering from diabetes mellitus are more prone to tendon injuries. Diabetic patients often have mechanically weaker tendons, characterized by poorly organized collagen fibers and disrupted extracellular matrix (ECM) [166].

Insulin-Like Growth Factor 1 (IGF-1) is a hormone that is associated with diabetes. Proper regulation of IGF-1 is crucial for maintaining healthy tendons, as it is involved in glucose uptake and promotes insulin sensitivity. Dysregulation of IGF-1 can led to insulin resistance and type 2 diabetes [167]. The effects of IGF-1 on tendon health are well documented. IGF-1 treatment in a rat model of AT exhibited anti-inflammatory effects, improved functionality, and faster tendon repair and also promoted collagen protein synthesis [168,169]. In another study, administration of IGF-1 to each patellar tendon significantly enhanced tendon collagen synthesis [170]. These studies highlight the importance of IGF-1 in stimulating collagen synthesis, which may in turn promote tendon repair.

Estrogen deficiency is also associated with an altered tendon structure and reduced muscle mass. Furthermore, lower estrogen levels can diminish collagen synthesis and increase the risk of tendon ruptures. This is particularly problematic in postmenopausal females, as estrogen levels drastically decrease after menopause. This reduction has been linked to a higher incidence of tendinopathy in females [171]. Therefore, hormone therapy is a viable treatment option for post-menopausal women. Menopausal hormone therapy (MHT) may reduce the likelihood of tendon abnormalities [172]. When combined with exercise, MHT showed more promising results and was associated with improvements in pain and function [171,172]. A similar effect of supplemental estrogen (SE) on tendon structure, synthesis, and biomechanical properties in post-menopausal women was observed. The SE group demonstrated increased tendon collagen synthesis and reduced tendon stiffness. Ultimately, SE was found to positively influence tendon morphology and biomechanical properties in post-menopausal women [173].

### 1.9. Stem Cells

#### 1.9.1. Mesenchymal Stem Cells (MSCs)

Mesenchymal stem cell (MSC) therapy is a safe and minimally invasive treatment with promising results in regenerative medicine. MSCs have demonstrated potential in improving pain, function, and radiological parameters in tendon disorders. Although the exact mechanism of action remains unclear, it is believed that MSCs differentiate into tenocytes and induce regenerative paracrine effect. In addition to their differentiation potential, MSCs also release a diverse array of cytokines, chemokines, and growth factors. Current evidence suggests that MSCs play a role in promoting tendon repair. For instance, the positive potential of MSCs for augmenting rotator cuff repair surgery and for patients with partial-thickness tears has been documented [174].

MSCs can be derived from various sources, including adipose tissue, bone marrow, the placenta, umbilical cord, and peripheral blood. Derivation from different tissue sources may lead to phenotypic variability (cellular heterogeneity), which could impart specific therapeutic benefits to MSCs. Future research should focus on developing methods to capture and expand specific MSCs that exhibit these properties [175]. Studies in animal models have supported the use of MSCs as a treatment for tendon disorders. Freshly thawed umbilical cord-derived mesenchymal stem cells (T-UCMSCs) were used for the treatment of full-thickness tendon defects. T-UCMSCs significantly improve the repair of rotator cuff tendons, as confirmed by macroscopic, histological, and biomechanical assessments [176].

Although limited human clinical studies exist, these findings indicate that MSCs have beneficial effects when used as a treatment. Intratendinous injection of the adipose-derived mesenchymal stem cells (ADMSCs) significantly improved shoulder function with no reported adverse effects. The mid-higher-dose group exhibited reduced shoulder pain, and arthroscopy confirmed a reduction in side defects [177]. In a separate study by Freitag and co-workers, ADMSCs were used to treat lateral epicondylitis (LET). Researchers observed that the combination of platelet-rich plasma (PRP) and ADMSCs resulted in successful tendon repair. Patients reported significant overall improvements with reduced pain scores, and subsequent follow-ups indicated that all patients were asymptomatic [178].

#### 1.9.2. Adipose-Derived Stem Cells (ADSCs)

Adipose-derived stem cells (ADSCs) are a subset of mesenchymal stem cells (MSCs) that can be derived from adipose tissue obtained via liposuction, aspiration, and excision. ADSCs can also be isolated from the stromal vascular fraction (SVF) through a series of steps, including washing, digestion, incubation, and removal of excess debris. ADSCs have advantages over bone marrow-derived MSCs (BMSCs), such as higher yield and greater proliferative activity. Furthermore, their role in regenerative medicine has become evident because of their potential in osteogenic, cartilage, myocardial, and nervous system regeneration [179].

ADSCs have been extensively studied in animal models, and it is increasingly evident that they play a therapeutic role in the treatment of tendinopathy. ADSCs have the potential to normalize collagen levels and promote regeneration. Oshita and co-workers evaluated the effects of ADSCs in a rat tendinopathy model and found that ADSC treatment promoted tendon repair in both the acute (4 weeks) and chronic (12 weeks) stages. Treatment with ADSCs helped normalize collagen levels and reduced overall degeneration [180]. In another study, the efficacy of human ADSCs (hADSCs) was assessed in a rat rotator cuff tear (RCT) model. The injection of hADSCs resulted in significant improvements in the tensile strength of the supraspinatus tendon and attenuated the progression of tendinopathy [181].

Extracellular vesicles (EVs) have also been used as paracrine modulators to promote tendon repair. Chen and co-workers evaluated the regenerative ability of ADSC-derived extracellular vesicles (ADSC-EVs). They first assessed the effects of ADSC-EVs in vitro and found that treatment with ADSC-EVs promoted tenocyte proliferation, migration, and upregulation of tendon markers. In a subsequent in vivo study using a rabbit Achilles tendon resection model, ADSC-EV treatment improved biomechanical properties, collagen synthesis, and maturation in resected tendons [182].

ADSCs can also promote tendon repair by reducing inflammation. In a study by Kokobu et al., treatment with ADSCs reduced inflammation and promoted tendon repair in a rat Achilles tendon (AT) model. Additionally, ADSC transplantation increased neovascularization by modulating hypoxic conditions and interleukin-1 beta (IL-1B) expression, resulting in lower rates of degeneration [183]. In another study by Shen and co-workers, connective tissue growth factor (CTGF) was used in combination with ADSCs to modulate inflammation and stimulate tendon regeneration in a canine flexor injury model. This combined treatment led to improved collagen production and reduced inflammation [184].

Although clinical studies are limited, ADSCs have shown positive outcomes. Lee et al. evaluated the efficacy and safety of allogeneic ADSCs for treating lateral epicondylitis (LET). The treatment resulted in decreased elbow pain, with no major adverse effects observed. Structural defects in pathological tendons were reduced, as observed via ultrasonography, and patients reported improvements in pain and performance scores throughout the study [129]. Khoury and co-workers also evaluated the effect of autologous ADSCs in LET treatment. Ultrasound-guided ADSC injection resulted in significant improvements in functional scores after one month of treatment, and structural repair of the tendon was observed using MRI. Overall, autologous ADSC treatment resulted in significant clinical improvement with no adverse effects [185].

#### 1.9.3. Tendon-Derived Stem Cells (TDSCs)

Tendon-derived stem cells (TDSCs) are pluripotent stem cells that have revolutionized tendon cell biology. TDSCs are considered an ideal cell type for tendon regeneration because of their proliferative and differentiation potential as well as their close resemblance to the tenocyte phenotype. TDSCs possess distinct traits such as a smaller cell body, larger nuclei, multipotency, and self-renewal capacity. Various factors are responsible for maintaining the multidirectional differentiation of TDSCs, including aging, growth factors, cytokines, genes, the microenvironment (extracellular matrix, ECM), and proper media. Although TDSCs are still novel in scientific research, they are emerging as an important tool in regenerative medicine [186].

Among these factors, aging may be the most frequently associated with stem cells. Aging can significantly decrease the differentiation capacity of TDSCs. In a study by Chen and co-workers, TDSCs isolated at different stages of development were compared and characterized. They found that TDSCs isolated in the first week exhibited better renewal, proliferation, and differentiation, highlighting the importance of using stem cells in earlier stages to maximize their potential [187].

As TDSCs represent a novel area of research, studies are primarily limited to animal tendinopathy models. In a study by Ni et al., TDSCs were used in a rat model of patellar tendon defects. The tendon defect was treated with a fibrin glue construct with or without TDSCs. The fibrin glue construct with TDSCs showed significant improvements in the early stages of tendon healing compared with constructs without TDSCs. This was indicated by (1) an increase in collagen production, (2) proper cellular and collagen alignment, and (3) superior biomechanical function in the regenerated tendon. This study supports the use of TDSCs for early recovery following tendon injury [188]. Thus, TDSCs may induce a stronger recovery response when used in combination with other treatments. This was investigated by Lui et al., who evaluated the effects of combining TDSCs, CTGF, and ascorbic acid in a rat patellar tendon model. The combinatorial treatment with TDSCs, CTGF, and ascorbic acid improved clinical outcomes and promoted earlier and better tendon repair. Tendon healing was further analyzed by histological, sonographic, ultrastructural, radiological, and biomechanical examinations, and significant improvements were observed [189].

The potential use of TDSC-derived exosomes (TDSC-Exos) has also been documented. Zhang et al. investigated the potential of TDSC-Exos in an in vitro study on tenocytes and found that TDSC-Exos promoted the proliferation and migration of tenocytes, thereby aiding tissue injury healing. They expanded their findings by using a rat Achilles tendon model, in which TDSC-Exos treatment induced anti-inflammation and promoted tendon healing. In conclusion, TDSC-Exos facilitated proper healing by inhibiting inflammation and reducing scar formation [190]. Similarly, Song et al. observed comparable results in vitro when TDSC-Exos were used. They evaluated the efficacy of TDSC-Exos in a rat patellar tendon defect model. TDSC-Exos were loaded onto photopolymerizable hyaluronic scaffolds and delivered to the defect site, improving performance in biomechanical testing and facilitating tendon repair [191].

Although autologous TDSCs may be a viable treatment for tendon-related disorders, clinical research on TDSCs is lacking, particularly when considering factors such as non-specific markers, heterogeneity, identification, aging, predisposition, and differentiation status. Further research is required to address these factors [192].

#### 1.9.4. Induced Pluripotent Stem Cells (iPSCs)

Induced pluripotent stem cells (iPSCs) are pluripotent stem cells generated from genetically engineered adult somatic cells. iPSCs are important because of their self-renewal and pluripotency potential. Since the discovery of iPSCs, various methods have been developed for their generation, including excision techniques, non-viral systems, and non-integrating viral vectors. The discovery of iPSCs was a major breakthrough because they resemble embryonic stem cells (ESCs) in many aspects. Although they have advantages, such as the elimination of ethical concerns, reduced immune rejection, cost-effectiveness, and the potential for personalized treatment, there are concerns surrounding mutations and genomic alterations that may accompany reprogramming. Consequently, the clinical use of iPSCs remains limited, and studies are ongoing to reduce DNA damage during reprogramming [193].

iPSCs can be used to repair injured tissues by transplanting appropriately programmed iPSCs to the site of injury. They have potential applications in treating various conditions, including musculoskeletal disorders, spinal injuries, and liver damage [194]. Owing to their ESC-like characteristics, iPSCs may induce a clinically positive response when treating tendon disorders. Komura and co-workers used tendon-specific reporter mice (SCX-EGFP) to generate iPSC-derived tenocyte-like cells (iPSC-TCs). The efficacy of iPSC-TCs was evaluated by transplanting them into injured Achilles tendons of mice. iPSC-TCs significantly improved tendon regeneration via a paracrine mechanism, resulting in well-aligned collagenous ECM and improved histological scores in the injured Achilles tendons following transplantation [195]. Nakajima and co-workers developed human iPSC-derived tenocyte-like cells (hiPSC-TCs) that strongly resemble primary tenocytes. The efficacy of hiPSC-TCs was assessed by transplanting them into a rat model of Achilles tendon rupture. Transplanted hiPSC-TCs expressed tendon collagen in host tissues two weeks post-transplantation, suggesting that hiPSC-TCs may induce a paracrine effect, promoting motor function recovery following Achilles tendon injury [196].

Tsutsumi and co-workers have generated a ‘Biotendon’ (tendon-like tissue) using iPSCs differentiated into MSCs and transfected them to MKX (iPSC-MSC-MKX). This Biotendon was subsequently transplanted into a mouse model of Achilles tendon rupture. The researchers observed that the transplanted ‘Biotendon’ significantly improved histological structures within the animal model, as indicated by well-aligned collagen fibers and enhanced mechanical properties compared to the control. The Biotendon may promote the recruitment and differentiation of tendon cells to reconstruct injured tendon tissue [197].

### 1.10. Epigenetic Regulators

Epigenetics plays an important role in the pathogenesis of various diseases, including cancers, cardiovascular diseases, neurological conditions, and musculoskeletal disorders. It regulates numerous processes, such as the recruitment and regulation of proteins via DNA methyltransferases, histone deacetylases, DNA replication and transcription, and the action of miRNAs on target genes. Despite their significance in the initiation, progression, and regulation of inflammation, the epigenetic mechanisms underlying tendon pathologies remain largely unexplored. Investigating the interactions between epigenetic processes and tendon-specific inflammatory genes may pave the way for novel therapeutic strategies to treat tendon disorders [198].

Genome-wide studies are instrumental in identifying changes in methylation that are associated with disease development and progression. Trella and co-workers conducted a study using a murine Achilles tendon (AT) model to identify epigenetic changes and identified five genes (Leprel2, Foxf1, MMP25, Igfbp6, and Peg12) that showed strong correlations in the AT model. These genes may provide insights into the underlying pathogenesis of tendinopathy [199]. In a genome-wide association analysis (GWAS) aimed at identifying polymorphisms linked to patellar tendinopathy (PT), Kim and co-workers highlighted two single nucleotide polymorphisms (SNPs) associated with PT pathogenesis, both located in the cytochrome c oxidase assembly factor 1 (COA1) gene. Studies elucidating the biological mechanisms linking COA1 with PT could be beneficial in designing therapeutic strategies [200].

Among epigenetic processes, the role of miRNAs in treating various disorders is well documented, and emerging research indicates their significance in tendon pathology. Watts and co-workers tested the efficacy of miR-29 in an equine model of superficial digital flexor tendon defect (SDFT). Treatment with miR-29 resulted in improved histological scores and tissue quality, and reduced levels of COL3, leading to enhanced tendon healing. miR-29 was found to mediate post-transcriptional regulation of collagen, resulting in the modulation of a collagen switch, a hallmark pathological feature of equine tendinopathy. Thus, the local delivery of miR-29 demonstrated a positive effect on tendinopathy [201].

Liu and co-workers evaluated the effects of miR-378a on tenogenic differentiation in a mouse model of patellar tendon defect. They found that miR-378a attenuated tenogenic differentiation both in vitro and in vivo, potentially negatively impacting extracellular matrix (ECM) production and tendon healing by suppressing the activity of TGFB2 [202]. Another study by Jun and co-workers explored the effects of miR-140-5p on tendinopathy and found that overexpression of miR-140-5p inhibited TL4 activity, which improved cell viability. The knockdown of TL4 by miR-140-5p enhanced tendinopathy in vitro [203]. Mao and co-workers investigated the efficacy of miR-205 inhibition and revealed that miR-205 inhibition resulted in increased cell proliferation, fibrosis, and tenocyte migration, suggesting that miR-205 may negatively regulate tendon injury repair, and that its pharmaceutical inhibition could serve as a therapeutic target for treating tendinopathy [204].

Chen and co-workers reported that miR-135 aids in osteogenic differentiation and acts as a negative regulator of TDSC senescence by targeting ROCK1. Overexpression of miR-135 in young TDSCs resulted in suppressed senescence, increased cellular proliferation, and enhanced migration, whereas its underexpression had the opposite effects. Normalizing miR-135 levels may facilitate recovery in aged tendons [205]. Therefore, it is evident that epigenetic mechanisms are involved in the pathogenesis of tendinopathy, although the precise mechanisms by which they regulate this process remain unclear. In summary, while currently established therapeutic interventions can provide symptomatic relief for tendinopathy, these therapies have certain limitations, which are outlined in Table 3.

However, there are still advances in the development of novel therapeutic approaches for promoting tendon healing. These therapies have been successful in promoting tendon regeneration in various types of tendinopathy. These therapies have been utilized to improve recovery in AT, RCT, PT, and LET, and are discussed in Table 4.

## 2. Discussion

Degenerative tendinopathy presents a significant therapeutic challenge owing to its multifactorial nature and variability in patient responses to treatment. Several factors have been identified as major contributors to the pathogenesis of tendinopathy, including aging, gender, and tendon overloading (Table S1). Although traditional approaches, such as exercise counseling (EC) and non-steroidal anti-inflammatory drugs (NSAIDs), have yielded positive clinical outcomes, such as reduced pain, their efficacy is largely limited to short-term benefits for most patients (Table 3 and Table 4).

In recent years, alternative therapies, including biologics, stem cells, and epigenetic regulation, have been investigated. These therapies have shown promising results but also exhibit limitations that must be addressed before they can be considered as optimal therapeutic alternatives (Table 2 and Table 3). Nevertheless, regenerative medicine, particularly the use of platelet-rich plasma (PRP) and stem cell-based therapies, has demonstrated considerable potential. PRP, which is rich in bioactive growth factors, has resulted in significant improvements in pain and function in various tendinopathies. Stem cell therapies, especially those involving mesenchymal stem cells (MSCs) and stromal vascular fractions (SVFs), have shown efficacy in preclinical and early clinical studies. However, the clinical translation of these findings faces challenges, including issues related to cell sourcing, scalability, and regulatory hurdles (Table 2 and Table 3).

Emerging regenerative therapies, such as stem cells and platelet-rich plasma (PRP), have shown promise in treating musculoskeletal conditions like osteoarthritis and tendinopathy. However, these therapies are often associated with considerable costs that limit their accessibility for many patients. For instance, PRP injections range from approximately USD 350 to USD 2815, imposing a significant financial burden [252]. The stem cell therapy market, divided into allogeneic and autologous stem cell therapies, highlights this financial strain further: autologous treatments, tailored to individual patients, are particularly costly. It is projected that the use of autologous cells will decline from 56% to 35% by 2029, largely due to these high preparation and treatment costs, making allogeneic stem cell therapy a more viable long-term investment [253].

When compared to conventional therapies like eccentric exercise or non-steroidal anti-inflammatory drugs (NSAIDs), regenerative options may provide added therapeutic benefits but struggle with cost-effectiveness. Conventional treatments, particularly eccentric exercise, maintain a well-established profile of cost-effectiveness and are more widely accessible to diverse patient populations [254]. Consequently, addressing the financial and accessibility challenges associated with PRP and stem cell therapies could enhance their feasibility as mainstream treatment options, ultimately providing patients with a broader, more inclusive range of therapeutic interventions.

Emerging therapies such as gene therapy and epigenetic modulation offer new avenues for treatment by targeting specific molecular pathways involved in tendon degeneration and repair. Modulation of gene expression through microRNAs (Table S2) or CRISPR-based approaches could potentially enhance tendon repair and mitigate fibrosis, as evidenced by preclinical models. Despite these advancements, several limitations of this study remain. Heterogeneity in patient populations, variability in disease severity, and the absence of standardized treatment protocols contribute to inconsistent clinical outcomes. Additionally, the high costs associated with regenerative therapies and need for long-term follow-up studies present significant challenges. 

## 3. Conclusions

Degenerative tendinopathy significantly impacts patients’ lives, and current treatments, while improving, show variable effectiveness. This study underscores the need to develop reliable and affordable therapeutic strategies. Advancements in regenerative medicine, including blood-derived products and cell-based therapies, have shown promise in promoting tendon healing. However, clinical data remain inconsistent, presenting a challenge for identifying universally effective treatments. Future research should focus on understanding the underlying mechanisms of tendinopathy and refining therapeutic approaches to provide consistent and long-lasting relief.

## 4. Future Directions

Future research should focus on conducting large-scale randomized controlled trials to validate the efficacy of emerging treatments and to develop biomarkers for early diagnosis and treatment monitoring to improve patient outcomes. Understanding the molecular mechanisms underlying tendinopathy will facilitate the development of targeted therapies that provide more consistent and long-lasting benefits. Collaborative efforts among researchers, clinicians, and regulatory bodies are essential to effectively translate these advancements into clinical practice. In conclusion, although traditional therapies remain crucial in managing tendinopathy, integrating regenerative medicine and emerging biotechnologies has the potential to significantly improve patient outcomes. Continued innovation and rigorous clinical validation are paramount for overcoming the current challenges and realizing the full therapeutic potential of these advanced treatments.

## Figures and Tables

**Table 2:** Short- and long-term benefits of cell-based therapies.

Therapy	Short-Term Benefits	Long-Term Benefits	Reported Complications
Platelet-Rich Plasma (PRP)	A single PRP injection in 83 tendons with non-insertional Achilles tendinopathy significantly improved VISA-A scores at six months, showing beneficial outcomes and a low complication rate, with no cases of Achilles tendon rupture [112].	In a two-year follow-up of 28 athletes with chronic patellar tendinopathy, three ultrasound-guided PRP injections resulted in significant improvements in VISA-P, VAS, and Lysholm scores. MRI showed enhanced tendon structure, with over half achieving complete normalization [113].	A 35-year-old male with patellar tendinopathy experienced localized swelling, erythema, and pain post-PRP injection, though no systemic inflammatory syndrome was present. The role of diabetes in this reaction is speculative based on this single case [114].
	Five patients with patellar tendinopathy showed improved VISA-P scores following a single PRP injection combined with rehabilitation, indicating the feasibility of PRP as an adjunct to physical therapy for degenerative patellar tendinopathy [115].	Forty-three patients with chronic proximal patellar tendinopathy demonstrated stable medium-term results, with consistent VISA-P score improvements at two months, six months, and four years post-PRP injection [116].	A 47-year-old female with knee pain experienced fever and severe pain 24 h after the third PRP injection, though the relationship between early exercise and the joint reaction remains unclear [117].
	In a study of 13 patients treated with ultrasound-guided PRP for tendinosis, nearly all reported symptom improvement at three months, with significant enhancement in GLRLM features [118].	Sixty-four non-athletes with lateral elbow tendinopathy treated with PRP showed better mean VAS scores at two years compared to corticosteroid treatment, indicating a sustained biological healing effect [119].	A 66-year-old male developed exuberant synovitis after a PRP injection following shoulder arthroscopy and subacromial decompression, likely due to platelet-rich growth factor treatment [120].
	Ninety-nine patients (47 in the PRP group and 52 in the corticosteroid group) showed superior PRP outcomes in VAS, ASES, and WORC scores at three months, though the advantage was not sustained at 12 months [121].	Eighty patients with lateral elbow tendinopathy treated with PRP demonstrated better outcomes compared to corticosteroids at three and 12 months post-injection, despite corticosteroids providing better short-term improvements at six weeks [122].	
	Twenty patients with chronic proximal patellar tendinopathy, randomized to receive either one or two PRP infiltrations, showed no significant differences in short-term outcomes, suggesting a second injection is unnecessary [123].		
Stem Cells	In a study of 26 patients with supraspinatus tendon tears, those treated with allogenic adipose tissue-derived mesenchymal stem cells (MSCs) combined with fibrin glue showed no significant benefit over saline for pain relief or shoulder function [124].	In a study of 43 patients with non-insertional Achilles tendinopathy, those treated with adipose-derived stromal vascular fraction (SVF) showed superior clinical outcomes to those treated with PRP, including better VAS, AOFAS, and VISA-A scores at 15- and 30-days post-treatment [125].	A study involving autologous MSCs for supraspinatus tendon repair was prematurely terminated due to post-operative complications in both the treatment and control groups, with high rates of re-rupture attributed to the scaffold (OrthADAPTTM) rather than the MSCs [126].
	In a study comparing bone marrow-derived MSCs (BM-MSCs) to PRP, both groups showed significant improvements in VISA-P scores at six months. However, BM-MSCs were superior in improving tendon structure [127].	Horses treated with adipose-derived MSCs for tendon disease showed significant reductions in inflammation and improved weight-bearing at 6- and 18-months post-injection, as indicated by lameness scores [128].	In a pilot study of allogeneic adipose-derived stem cells (allo-ASCs), injections for lateral epicondylitis, mild swelling, and joint effusion were reported in some patients but resolved without intervention. No immunologic rejection was detected [129].
	Nine horses with tendon lesions treated with adipose-derived MSCs showed no significant improvement over controls, highlighting the need for further research on MSC efficacy in tendon repair [130].	Twelve participants with lateral epicondylitis treated with allo-ASC injections reported decreased pain and improved tendon structure at 52 weeks, marking the first clinical use of allo-MSCs in chronic tendinopathy treatment [129].	
Adipose Stromal Vascular Fraction (SVF)	In a controlled study of 56 patients with non-insertional Achilles tendinopathy, those treated with SVF showed faster and greater improvements in VAS, AOFAS, and VISA-A scores compared to PRP-treated patients, with significant differences observed at 15 days post-injection [131].	A case study of a 27-year-old patient with chronic insertional Achilles tendinopathy demonstrated continuous clinical improvement following an SVF injection, with significant progress observed at 1 month and sustained throughout the 12-month follow-up [132].	In a study of 20 osteoarthritis patients treated with autologous concentrated adipose tissue, one patient experienced tendonitis-like swelling two months post-procedure, and two patients withdrew from the study due to persistent pain, opting for knee replacement [133].
	In a trial of 43 patients with non-insertional Achilles tendinopathy, both SVF and PRP treatments significantly reduced pain, with SVF demonstrating faster recovery and better outcomes in tendon thickness and blood flow [134].	Fourteen patients with chronic insertional patellar tendinopathy receiving autologous adipose-derived stem cell injections showed significant pain reduction and improved sports activity function at 3, 6, and 12 months post-treatment [135].	

**Table 3:** Advantages and drawbacks of tendinopathy treatments.

Treatment	Advantages	Disadvantages
Eccentric training (EC)	EC shows the best result when it is used in combination with other therapies [11]. Nevertheless, EC as a mono-treatment was found to increase collagen 1 synthesis in human ATS [68]. Furthermore, EC shows reduced pain scores for chronic AT [71].	Previous studies have not examined the long-term benefits of using EC as a mono-treatment [11]. Delayed onset muscle soreness may accompany EC [206].
Diet and Nutrition	Nutraceuticals such as VC have been shown to promote collagen production, and when used in combination, can significantly enhance tendon health and metabolism [78,79].	Although collagen seems to be beneficial in the treatment of tendinopathy, conclusions about optimal dosage, timing, duration, and type of collagen supplementation require further studies [207]. Although nutraceuticals are known to benefit tendon health, improper diet, i.e., a high fat diet, can negatively affect the tendon by reducing cellular proliferation and decreasing collagen synthesis [208].
Extracorporeal Shockwave therapy (ESWT)	Previous studies have suggested ESWT shows optimum results when combined with other therapies. ESWT in combination with EC significantly improved functional scores [24]. Similarly, when combined with PRP, it helped to promote growth factor production [78].	Patients treated with moderate- to high-energy ESWT have reported of pain following treatment [209].
Anti-inflammatory treatment (NSAIDs, GCs)	The majority of the previous studies suggest anti-inflammatory drugs such as NSAIDs and GCs work best when used in combination with other therapies. NSAIDs have been suggested to help in tendon healing by improving collagen synthesis [210]. Furthermore, when GCs were used in combination with EC, patients reported positive follow-up for 24 months [102].	There is conflicting evidence to support the efficacy of NSAIDs and GCs [101].
Platelet-rich plasma (PRP)	As it is derived from a patient’s own blood, it is a safe treatment with minimal chances of local infection [137]. Furthermore, PRP is rich in growth factors, which may help to promote COL1, which is a major component of the tendon [140].	Prior studies have suggested that it may not provide any significant improvement over other conventional treatments [211]. Furthermore, 2–3 injections of PRP are advised to observe significantly positive clinical outcomes [212]. Optimizing dosage of PPR is, therefore, a rising concern because PRP treatment is extremely costly [213].
Stromal Vascular Fraction (SVF)	SVF treatment promoted COL1 synthesis and showed improvement in structure and functionality of the tendon [155]. Furthermore, because of its efficacy and safety, it may be a good alternative to anti-inflammatory drugs like NSAIDs [156].	SVF may induce an inflammatory response. The process involved in inducing this response remains unknown [214]. As SVF constitutes a heterogenous population of cells with progenitor potency, SVF may cause risks of neoplasm and unwanted cell differentiation [215].
Stem cells	MSCs, when used in combination with PRP, promoted COL1 synthesis in LET patients [178]. ADSCs were used for treatment of LET. ADSCs aided in relieving pain and improved functionality in patients [129]. TDSCs have been utilized to promoted early tendon healing in a rat tendinopathy model [188]. Human iPSCs were successfully transplanted in a rat model of AT. iPSCs helped to improve overall biomechanical functionality of the tendon [196].	As there is no optimized protocol for optimal stem cell harvesting, previous studies have reported inconsistent findings [216]. Some prior studies suggest ADSCs may activate certain signals and inadvertently promote oncogenesis [217]. The preparation of IPSCs is a complex problem requiring directed differentiation and minimizing mutation and malignant transformation in vitro [218].
Epigenetics/miRNA	Genome-wide studies may help in identifying genome-wide changes in methylation associated with tendinopathy pathogenesis [199,200].	Identifying epigenetic marker is difficult because of large gene lists, few comparative studies, and conflicting studies [219]. Genetic discrimination and misuse of epigenetic information is a rising concern in the scientific world [220].

**Table 4:** Treatment response in various tendinopathies.

Findings	Achilles Tendinopathy (AT)	Rotator Cuff Tendinopathy (RCT)	Patellar Tendinopathy (PT)	Lateral Elbow Tendinopathy (LET)
Eccentric training (EC)	EC helped to decrease tendon thickness and normalized tendon structure [221]. A recent meta-study suggests using EC can provide positive outcomes when treating AT [222].	Eccentric exercise may provide long-term benefits for patients with RCT [223].	EC improved tendon resistance in PT [224], making EC a safe and tolerable treatment for PT [225].	EC was shown to improve functionality and provided pain relief in LET [226].
Diet and nutrition	Vitamin C (VC) treatment was effective in pain management for patients with AT [80].	VC supplementation improved tendon healing in rotator cuff tendon repair [227]. Pain management in RCT can be better managed by using supplements that aid in collagen organization in combination with physiotherapeutic interventions [228,229].	Patients reported reduced pain and better performance after following a VC and gelatin-rich diet [228,229]. A supplementation using β-Hydroxy β-methyl butyric may enhance muscular performance in PT patients [230].	No prior findings.
Extracorporeral shockwave therapy (ESWT)	ESWT showed a reduction in pain and inflammation compared to mesotherapy for treatment of AT [231]. ESWT also provided region-targeted benefits. In one study, ESWT showed effectiveness when treating mid-portion AT [232].	Low-energy ESWT was effective in providing short-term benefits in supraspinatus tendinopathy [233]. In a recent clinical trial, high-energy ESWT reduced pain and improved range of motion and quality of life in RCT [234].	A modified ESWT provided immediate pain relief to PT patients [235].	ESWT is superior to laser therapy, and five sessions were enough to significantly improve overall functionality and grip strength in LET [236]. ECST provided improve functional performance and grip strength when compared to a placebo [237].
Anti-inflammatory treatment (NSAIDs, GCs)	In a 10 year follow up study, one or two injections of GCs was found to be safe and showed positive outcomes [238]. GC injection in combination with physiotherapy showed significant symptomatic improvement in AT [156].	Following rotator cuff repair, ibuprofen did not increase the risk for tendon rupture and significantly improved pain reduction [239].	GCs, EC, and heavy slow resistance training showed improvement in pain and functionality up to 6 months in PT [240].	GCs and acupuncture are good treatment options, providing short-term benefits for LET [241].
Platelet-rich plasma (PRP)	Autologous plasma injection in combination with exercise and ultrasonography clinically improved AT. [242]. A recent meta-study reports a significant increase in VAST-A score [243]. PRP is a safe and effective treatment capable of ameliorating pain and improving functionality in both chronic and acute AT [244].	A single injection of PRP was sufficient to reduce pain and improve functionality in RCT [245]. PRP for RCT is safe and provides long term improvements in shoulder pain and function [246].	Mono-PRP was a more practical treatment compared to high volume image-guided injection (HVIGI) combined with physiotherapy for PT [247].	A single injection of PRP provides long-term benefits compared to corticosteroid injections [248].
Stromal vascular fraction (SVF)	SVF showed significant functional improvement just one month post-treatment [132]. A single injection of SVF provided pain relief and was superior to PRP in terms of providing a quicker response [125].	In a rat model, SVF showed a 40% reduction in fibrosis compared to the control following rotator cuff tear and repair [249]. SVF administration yielded positive results in healing RCT in rabbits [250].	No prior studies	Autologous adipose SVF-ASC provided overall improvement in pain and performance [129].
Stem Cells	BM-MSC sheets were administered in RAT AT and showed significant improvements in biomechanical properties [251]. ADSC promoted neovascularization in a rat model of acute AT [204]. ADSC-EV promoted collagen 1 synthesis, cellular proliferation, and migration in a rabbit model of AT [205]. TDSC-Exos promoted tendon healing by promoting tenocyte proliferation and migration [195]. Grafted IPSC-Tenocyte promoted recovery post-Achilles tendon rupture [221].	ADMSCs showed significant improvement in shoulder pain for rotator cuff patients [177]. hADSC promoted tensile strength and lower inflammatory cells in a rat model of RCT [182].	In a rat patellar tendinopathy model, TDSCs promoted early tendon healing and increased collagen synthesis [188]. TDSC-CTGF-Ascorbic acid improved the clinical outcome and showed enhanced proliferation and cellular survivability [189]. BM-MSCs in combination with rehabilitation showed greater improvement in tendon structure than PRP and helped to reduce pain in PT [127].	A combination of PRP and MSCs promoted regenerative healing for LET patients [178]. Allogenic ADSCs showed improvement in pain and functionality in LET [129].

## Data Availability

Not applicable.

## Supplementary Materials

The following supporting information can be downloaded at https://www.mdpi.com/article/10.3390/ijms252111846/s1 [255,256,257,258,259,260,261,262,263,264,265,266,267,268,269,270].
