# Supplementary material for: Advancements in Therapeutic Approaches for Degenerative Tendinopathy: Evaluating Efficacy and Challenges
Source: Int J Mol Sci. 2024 Nov 4;25(21):11846. doi: 10.3390/ijms252111846 (PMC11545934; doi:10.3390/ijms252111846)
Supplement: Supplementary file 1 [file ijms-25-11846-s001.zip › ijms-3220562-supplementary.pdf]

Table S1. Treatment association with age and gender.

Therapy	Age	Gender
Eccentric Training (EC)	EC was effective even more so than resistance training for older adults. Suggesting, EC may be overall more beneficial for older adults regardless of age [255].	Following EC women showed less muscle inflammation compared to men [256].
Diet and Nutrition	Proper diet and nutrition and following healthy habits improves quality of life and promotes healthy aging [257].	Sex specific diet especially for female must exist in part due to difference in sex hormone concentration of both genders [258].
Extracorporeal shock-wave therapy (ESWT)	ESWT is considered a safe and effective treatment for both young and adult patients [259].	ESWT as a mono treatment relieved pain in males more effectively than in females [260].
Non-Steroidal anti-inflammatory drugs (NSAID)	NSAID use has been implicated in increasing elderly patient admission in hospital. The use should be monitored carefully [261].	Anti-inflammatory drugs induce a varying response based on gender. Giving rise to the idea personalized medicines should be an integral part for future research [262].
Platelet rich plasma (PRP)	Age impacted the levels of Growth factors (GFs) in PRP. As expression of certain GFs was higher in younger patients [263].	Certain GFs were more highly expressed in females compared to males [263].
Stromal Vascular Fraction (SVF)	Aging influences the cellular yield from SVF. As lowest yield was obtained from older patients [264].	In mice, male SVF expressed high levels of CD34, leucocyte compared to females [265].
Stem cells	Stem cell transplantation onto an older adult may be problematic because of age dependent defects [266].	Male and female derived stem cells have shown variability in activity as evidenced by studies on animal models/humans [267].
Epigenetic regulation	Aging is accompanied by a number of notable changes including global heterochromatic reduction, histone methylation silencing, miRNA activity and altered post translational modifications [268].	Study reports significant differences in DNA methylation between men and women. Certain methylated loci also exhibited differential gene expression [269].

Table S2. Characteristics of miRNAs in tendinopathy.

miRNA	Effects	Mechanism
miR-29	Improved histological scores, tissue quality, and reduced COL3 levels, leading to improved tendon healing [201].	Post-transcriptional regulation of collagen, helping to reduce overall COL3 levels [201].
miR-378a	Attenuated tenogenic differentiation in vitro and in vivo, negatively impacted ECM production and tendon healing [202].	Suppresses the activity of TGFB2 [202].
miR-140-5p	Improved cell viability and tendinopathy in vitro [203,270].	Inhibits activity of TL4 [203,270].
miR-205	Increased cell proliferation, fibrosis, and tenocyte migration [204].	Acts on tenocytes through MeCP2 [204].
miR-135	Aids in osteogenic differentiation, acts as a negative regulator of TDSC senescence, promotes cellular proliferation and migration [225,270].	Targets ROCK1. Overexpression suppresses senescence and enhances cellular proliferation; under-expression has the opposite effects[225,270].
